# Identification of nucleolar protein No55 as a tumour-associated autoantigen in patients with prostate cancer

**DOI:** 10.1054/bjoc.2000.1365

**Published:** 2000-08-17

**Authors:** A Fosså, R Siebert, H-C Aasheim, G M Mælandsmo, A Berner, S D Fosså, E Paus, E B Smeland, G Gaudernack

**Affiliations:** Departments of Immunology, Tumour Biology, Pathology, Medical Oncology, Central Laboratory, The Norwegian Radium Hospital, Montebello, Oslo, 0310, Norway; Department of Human Genetics, University of Kiel, Schwanenweg 24, Kiel, 24105, Germany

**Keywords:** SEREX, prostate cancer, No55

## Abstract

Four different genes were identified by immunoscreening of a cDNA expression library from the human prostate cancer cell line DU145 with allogeneic sera from four prostate cancer patients. A cDNA encoding the nucleolar protein No55 was further analysed and shown to be expressed at the mRNA level in several normal tissues, including ovaries, pancreas and prostate and in human prostate cancer cell lines PC-3, PC-3m and LNCaP. By reverse transcriptase/polymerase chain reaction, expression of No55 was several-fold higher in two out of nine prostate cancer primary tumours and two out of two metastatic lesions, compared to normal prostate tissue. Antibodies to No55 were detected in sera from seven out of 47 prostate cancer patients but not in sera from 20 healthy male controls. Sequence analysis of the No55 open reading frame from normal and tumour tissues revealed no tumour-specific mutations. The No55 gene was located to chromosome 17q21, a region reported to be partially deleted in prostate cancer. Considering the immunogenicity of the No55 protein in the tumour host, the expression profile and chromosomal localization of the corresponding gene, studies evaluating No55 as a potential antigen for immunological studies in prostate cancer may be warranted. © 2000 Cancer Research Campaign


British Journal of Cancer (2000) 83(6), 743–749
© 2000 Cancer Research Campaign
doi: 10.1054/ bjoc.2000.1365, available online at http://www.idealibrary.com on IDEAL®

### Identification of nucleolar protein No55 as a tumour-associated autoantigen in patients with prostate cancer

**A Fosså[1], R Siebert[6], H-C Aasheim[1], GM Mælandsmo[2], A Berner[3], SD Fosså[4], E Paus[5], EB Smeland[1] and G Gaudernack[1]**

Departments of[1] Immunology, [2]Tumour Biology, [3]Pathology and [4]Medical Oncology and [5]Central Laboratory, The Norwegian Radium Hospital, Montebello, 0310 Oslo, Norway; [6]Department of Human Genetics, University of Kiel, Schwanenweg 24, 24105 Kiel, Germany

**Summary** Four different genes were identified by immunoscreening of a cDNA expression library from the human prostate cancer cell line DU145 with allogeneic sera from four prostate cancer patients. A cDNA encoding the nucleolar protein No55 was further analysed and shown to be expressed at the mRNA level in several normal tissues, including ovaries, pancreas and prostate and in human prostate cancer cell lines PC-3, PC-3m and LNCaP. By reverse transcriptase/polymerase chain reaction, expression of No55 was several-fold higher in two out of nine prostate cancer primary tumours and two out of two metastatic lesions, compared to normal prostate tissue. Antibodies to No55 were detected in sera from seven out of 47 prostate cancer patients but not in sera from 20 healthy male controls. Sequence analysis of the No55 open reading frame from normal and tumour tissues revealed no tumour-specific mutations. The No55 gene was located to chromosome 17q21, a region reported to be partially deleted in prostate cancer. Considering the immunogenicity of the No55 protein in the tumour host, the expression profile and chromosomal localization of the corresponding gene, studies evaluating No55 as a potential antigen for immunological studies in prostate cancer may be warranted. © 2000 Cancer Research Campaign

**Keywords**: SEREX; prostate cancer; No55

One aim of tumour immunology continues to be the definition of antigens that are expressed in tumour cells and elicit immune responses in the autologous host (Kawakami and Rosenberg, 1997; Old and Chen, 1998). Using specific cytotoxic T-lymphocytes (CTL) and cultured tumour cells from individual patients, several such antigens have been identified, particularly in malignant melanoma (Kawakami and Rosenberg, 1997). These antigens are attractive candidates for molecularly defined vaccine-based immunotherapy of cancer. However, the direct definition of antigens recognized by CTLs is technically demanding and not feasible for many common types of malignancies.

SEREX (serological identification of tumour antigens by expression cloning) was introduced by Sahin et al (1995) as an alternative approach to identify antigens recognized by autologous serum antibodies. Basically, a cDNA expression library is constructed from tumour material and screened with the patient's serum, thus enabling cloning of genes encoding proteins which elicit B-cell responses. The validity of the approach for identification of tumour antigens giving rise to cell-mediated immune responses as well was exemplified by finding previously known CTL antigens among the first antigens isolated by SEREX (Sahin et al, 1995). Additionally, SEREX-defined antigens have later been shown to elicit T-cell responses (Jager et al, 1998).

SEREX has been used to screen expression libraries from various malignancies, including renal cell carcinoma (Sahin et al, 1995; Scanlan et al, 1999), Hodgkin's disease (Sahin et al, 1995), malignant melanoma (Chen et al, 1998; Sahin et al, 1995), adult T-cell leukaemia/lymphoma (Itoh et al, 1999), oesophageal squamous cell carcinoma (Chen et al, 1997), lung (Gure et al, 1998) and colon cancer (Scanlan et al, 1998). Variations of the original method include screening of subtracted testis cDNA libraries or libraries made from tumour cell lines (Chen et al, 1998; Itoh et al, 1999; Tureci et al, 1998). Over 1000 antigens belonging to several categories have been identified, including differentiation antigens, mutational antigens, overexpressed antigens and the so-called cancer/testis family of tumour antigens (Old and Chen, 1998). The latter category consists of genes or gene families that are uniquely expressed in normal testicular tissue and in malignant tumours. The high number of tumour antigens identified provide ample evidence for the recognition of cancer by the host immune system. However, the high number of antigens also necessitates thorough characterization of the identified genes to establish a potential role in cancer biology and immunotherapy.

The present paper describes the application of SEREX to screen for immunogenic tumour antigens in prostate cancer. The original method was modified to include a cell line derived cDNA library and sera from allogeneic patients with early and late stages of the disease to potentially increase the yield of antigens common to a larger proportion of prostate tumours. Among four genes identified, the gene encoding nucleolar protein No55 was further characterized in terms of expression pattern, serological response in prostate cancer patients and chromosomal localization.

*Received 23 November 1999*
*Revised 9 May 2000*
*Accepted 8 June 2000*

*Correspondence to:* A Fosså

### MATERIALS AND METHODS

### Patient sera, biopsy material and cell lines

The study was approved by the ethics committee for health region II, Oslo, Norway. Sera from patients with histologically verified prostate cancer and male normal volunteers were provided by the Central Laboratory, The Norwegian Radium Hospital (NRH). Biopsies from normal prostate tissue ($n$ = 5), primary prostate tumours ($n$ = 6) and metastatic lesions ($n$ = 2) were provided by the Department of Medical Oncology, NRH. Tissue areas adjacent to the material used for mRNA extraction were analysed for malignant cells by light microscopy of haematoxylin/eosin-stained paraffin sections. Matched cDNA preparations from primary tumour and normal prostate tissue of three individual patients were purchased from Clontech Laboratories, Palo Alto, CA, USA.

The following cell lines from human malignancies were used: prostate cancer: DU145 (ATCC HTB-B1), LNCaP (ATCC CRL-1740), PC-3 (ATCC CRL-1435) and PC-3m (provided by Dr IJ Fidler, MD Anderson Cancer Center, Houston, Texas, USA); Cervix cancer: HeLa (ATCC CCL-2); Pancreatic cancer: Capan-1 (ATCC HTB-79). Cell lines from lymphoid malignancies (Reh, BV173, Tom1) have been described recently (Rosok et al, 1999).

### cDNA library construction and immunoscreening

Total RNA was extracted from DU145 cells using the acid guanidinthiocyanat method (Chomczynski and Sacchi, 1987) and mRNA isolated with oligo-dT-Dynabeads (Dynal, Oslo, Norway). The cDNA library was constructed in the λZAP expression vector (Statagene, LaJolla, CA, USA). The cDNA library had $1.5\times10^6$ primary recombinant clones and was amplified once before screening.

Sera from one patient with metastatic disease (MD1) and three patients with localized disease (LD1–3) were used for immunoscreening as described by Gure et al (1998). Briefly, sera diluted 1:10 were extensively absorbed against bacteria transfected with wild type λZAP phage, further diluted to a final titre of 1:500 or 1:100 and a minimum of $4 \times 10^5$ recombinant plaques screened with each dilution. After washing, the filters were incubated with alkaline phosphatase conjugated goat anti-human IgG and immunoreactive plaques visualized by incubation with 5-bromo-4-chloro-3-indolyl-phosphate and nitroblue tetrazolium.

Positive phages were subcloned until homogeneity, converted to plasmid clones in pBluescript using the Ex-Assist helper phage (Stratagene) and sequenced by means of BigDye™ Terminator Cycle Sequencing (Perkin Elmer Applied Biosystems, Warrington, UK) on an ABI Prism™ 310 Genetic Analyser (Perkin Elmer). DNA and protein sequences were analysed using DNASIS for Windows 2.1 (Hitachi Software Engineering America, San Bruno, CA, USA) and compared to sequences in the GenBank and other public databases using the BLAST program.

### Cloning of No55 open reading frame (ORF) and rapid amplification of cDNA ends (RACE) by polymerase chain reaction (PCR)

The ORF of the No55 gene was amplified from normal testis Ready-Marathon-cDNA (Clontech) and a metastatic lesion of patient MD1 by PCR. Primers No55ORFf (5′-TCCGGAGAGC-CGGCGGCGCGGCGG-3′) and No55ORfr (5′-CACCAGGCT-TCCCAAGCTTGAGCGGTGTG-3′) were designed to cover the complete ORF. PCR products were cloned into the pGEM-T® Easy vector (Promega, Madison, WI, USA) and the ligation mixture used to transform competent *Escherichia coli* DH10B cells (Life technologies, Rockville, MD, USA). Four clones containing the desired PCR fragment were sequenced from each experiment.

RACE PCR was performed using cDNA from DU145 ligated into λZAP, but not in vitro packaged into phage particles, with primers T7 (5′-GTAATACGACTCACTATAGGGC-3′) and No55ORFr. PCR products were analysed by gel electrophoresis and the longest DNA fragments cloned in *E. coli* DH10B cells as above. Twelve clones with the longest PCR fragments were sequenced from their 5′ ends.

### Expression analysis of No55 in tissues and cell lines

Northern analysis of No55 in normal tissues was performed using commercially available blots (Clontech). For expression studies in cell lines, 10 μg total RNA separated by formaldehyde/agarose gel electrophoresis and transferred to nitrocellulose membranes. Hybridization was performed with a $^{32}$P-labelled 1530 bp fragment covering the complete ORF of the No55 gene. Standardization was achieved using a $^{32}$P-labelled fragment of β-actin cDNA (Rosok et al, 1999). Quantification was performed on a Storm 840 Phosphorimager using ImageQuant 5.0 (Molecular Dynamics GmbH, Krefeld, Germany).

Expression of No55 in tumour biopsies was analysed by reverse transcriptase/polymerase chain reaction (RT/PCR). mRNA was extracted using the mRNA-Quick-Prep Kit (Amersham-Pharmacia-Biotech, Oslo, Norway) and treated with RNase-free Dnase I (Boehringer-Mannheim, Mannheim, Germany). cDNA synthesis was performed with First-Strand cDNA Synthesis Kit (Amersham-Pharmacia-Biotech). For one set of experiments, primers No55961f (5′-CCAGCTACATGCTCTTCGACCCCA-3′) and No55ORFr were used to amplify a 408 basepair (bp) fragment from the 3′ end of the No55 ORF. A second set of experiments used the primers No55ORFf and No55ORFr covering the complete ORF. The number of cycles (34 and 30, respectively) was determined in preliminary experiments to be in the logarithmic phase of the PCR. After agarose gel electrophoresis PCR products were blotted to nitrocellulose for hybridization with a $^{32}$P-labelled fragment covering the 3′ end of the No55 ORF. Quantification was performed on a Storm 840 Phosphorimager as above. The amount of cDNA used per reaction was standardized according to the expression level of β-actin with primers β-actinf (5′-GAGCAAGAGAGGCATCCTCAC-3′) and β–actinr (5′-TGCCTCAGGGCAGCGGAACCG-3′) or human ribosomal protein S9 using commercially available primers (Clontech).

### Serological analysis for anti-No55 antibodies

Analysis of anti-No55 IgG antibodies in sera from patients with prostate cancer and healthy male controls was performed using a plaque assay (Gure et al, 1998). Recombinant phages encoding No55 and irrelevant phages were plated on LB-agar plates in a 1:5 ratio. Filters were hybridized to preabsorbed serum diluted 1:100 and positive plaques visualized as above. For recording as a positive result, positive and negative plaques in approximately a 1:5 ratio were required. In some positive cases incomplete circles were seen representing positive plaques in direct contact with negative plaques. Sera giving uniform faint staining of all plaques or completely negative filters were recorded as negative.

### Chromosomal localization of the gene encoding No55

Radiation hybrid mapping was performed with primers No55f (5′-TAATCGTAGCTGAGGCAG G-3′) and No55r-1 (5′-CAGAGA-

 *© 2000 Cancer Research Campaign*

CATAGAAGTGGCAGG-3′) by means of the Stanford G3 Panel (Research Genetics, Huntsville, AL, USA). Map localization was calculated on the Stanford Human Genome Center RHServer (*http://www-shgc.stanford.edu*). Additionally, the human RPCI1,3–5 PAC libraries were screened by PCR using the same primer pair. Pools for this screening were obtained from the German Resource Center (Berlin, Germany). Cytogenetic assignment of PAC clone 228H19 derived from the PAC library screen was performed by FISH using biotinylated PAC DNA as probe and subsequent R-banding as described recently (Harder et al, 1997).

### RESULTS

### Immunoscreening of the DU145 cDNA library

The DU145 cDNA expression library was screened with sera from four prostate cancer patients, and the results of immunoscreening are shown schematically in Table 1. cDNA inserts were grouped based on restriction mapping, partial sequencing and DNA–DNA hybridization. All sequences identified were cDNAs of known human genes. Serum from patient MD1 reacted with 12 plaques, representing two independent cDNA clones. Eleven of these clones contained the full-length cDNA of TDP-43, a human DNA binding protein known to be widely expressed in normal tissues (Ou et al, 1995). The last clone was derived from the mRNA of the No55 gene (Ochs et al, 1996). Sera from the three patients with localized disease (LD1, LD2 and LD3) reacted with 1, 17 and 0 plaques, respectively. For patient LD1, the only positive clone contained the full-length cDNA insert of Drebrin, an actin-binding protein known to be differentially expressed in neuronal development (Toda et al, 1993). The 17 plaques from screening with serum from patient LD2 originated from two different genes, No55 and the recently identified regulatory subunit of serine/threonine protein phosphatase 4 ($PP4_R$) (Kloeker and Wadzinski, 1999). Northern blot analysis showed $PP4_R$ to be broadly expressed in human tissues with predominant expression in testis, peripheral blood leukocytes and spleen (Figure 1). The TDP-43, Drebrin and $PP4_R$ cDNA clones were not analysed further.

### Sequencing

Further work focused on the 18 phage clones harbouring No55 cDNAs. The two clones with the longest cDNA inserts (LD2–3 and LD2–14) were sequenced and compared to the No55 sequence from the human bladder carcinoma cell line T24 (Table 1). Both

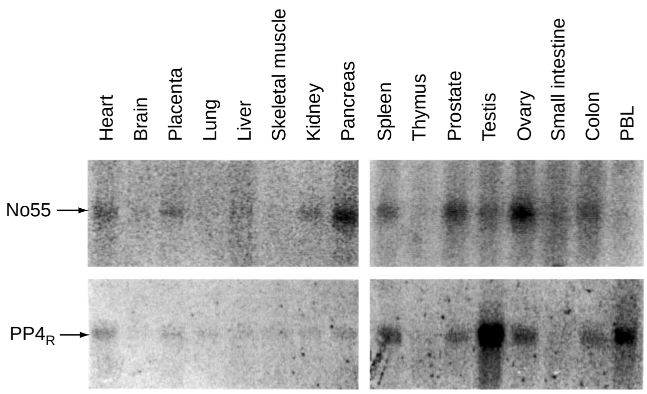

**Figure 1** Northern blot analysis in normal human tissues of nucleolar protein No55 (top panel, transcript length 2.6 kb) and the regulatory subunit of serine/threonine protein phosphatase 4 ($PP4_R$, lower panel, transcript length 3.6 kb). PBL = peripheral blood leukocytes cDNA clones terminated at position 2005 of the T24 sequence, probably as a result of hybridization of the oligo-dT adaptor primer used for first-strand synthesis to an internal 18 nucleotide adenine repeat. Compared to the T24 sequence, clone LD2–14 contained 43 additional nucleotides in the 5′ untranslated region (5′UTR). In both DU145 derived clones a base substitution (A→G) was detected at position 557 (one being A of ATG initiation codon), resulting in an amino acid substitution (Gln→Arg) at position 186 of the predicted primary structure of the No55 protein. A second substitution (G→C) was detected in both DU145 clones at position 1345, just 3′ of the TGA stop codon.

A 5′RACE-PCR approach was used to establish the extent of the 5′UTR not covered by the clones from the immunoscreening of the DU145 library. Twelve overlapping cDNA clones were sequenced at their 5′ ends and the longest insert covered an additional 73 nucleotides compared to the T24 sequence. The 5′UTR did not contain an ATG initiation codon or stop codons in any of the three possible reading frames. The complete sequence of the No55 mRNA from DU145 has been deposited to EMBL Genbank (accession number AJ250583).

To explore the possibility of sequence mutations as the reason for a serological response to No55, the complete ORF was sequenced both in normal testis tissue pooled from four caucasian males and in a metastatic lesion of the seroreactive patient MD1. In both cases, four PCR products were sequenced with an average of one random PCR artifact per clone. In testis, clones corresponding both to the sequence at nucleotide position 557 of T24

**Table 1** Results of immunoscreening of the DU145 expression cDNA library with sera from four prostate cancer patients

| Patient | Screening titre | Clones screened (*n*) | Positive clones (*n*) | Gene identified/ Accession no. | Comments |
|---|---|---|---|---|---|
| MD1 | 1:500 | $1 \times 10^6$ | 11 | TDP-43/U23731 | TAR DNA binding protein, widely expressed in normal tissues |
| | | | 1 | No55/U47621 | Nucleolar protein 55, see text |
| LD1 | 1:500 | $5 \times 10^5$ | 0 | | |
| | 1:100 | $4 \times 10^5$ | 1 | Drebrin/NM_004395 | Actin binding protein, differentially expressed in neuronal development |
| LD2 | 1:500 | $5 \times 10^5$ | 8 | No55/U47621 | Nucleolar protein 55, see text |
| | 1:100 | $4 \times 10^5$ | 8 | No55/U47621 | |
| | | | 1 | $PP4_R$/AF111106 | Regulatory subunit of serine/threonine phosphatase 4, widely expressed in normal tissues |
| LD3 | 1:500 | $5 \times 10^5$ | 0 | | |
| | 1:100 | $4 \times 10^5$ | 0 | | |

*© 2000 Cancer Research Campaign*

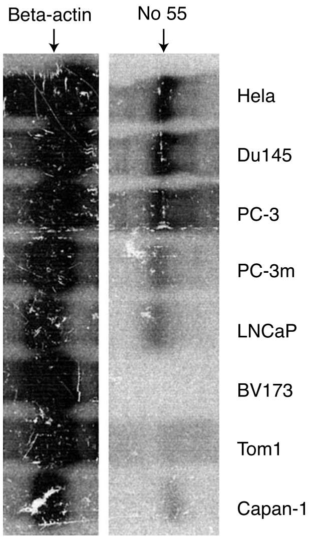

**Figure 2** Northern blot analysis of nucleolar protein No55 (right panel) in cell lines from prostate cancer (Du145, PC-3, PC-3m, LNCaP). Expression in HeLa (cervix cancer), BV173 and Tom1 (lymphoid malignancies) and Capan-1 (pancreatic cancer) are shown for comparison. β-actin expression as control of RNA quality and loading (left panel)

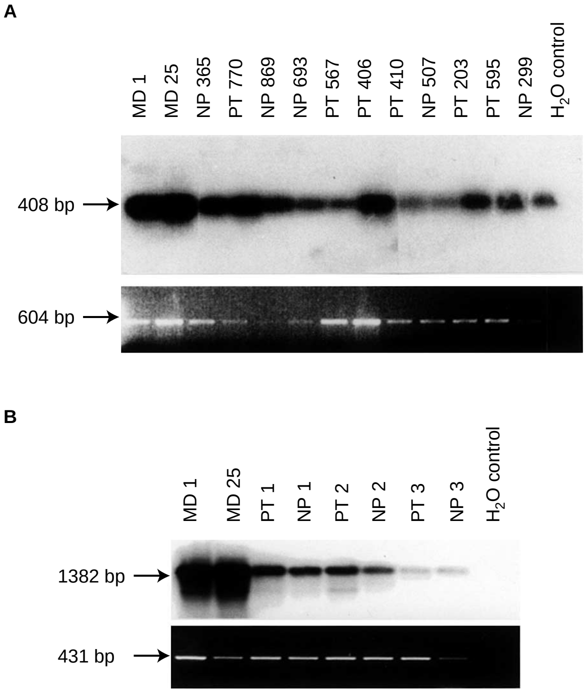

**Figure 3** Expression of nucleolar protein No55 in biopsies from normal prostate (NP), primary prostate tumours (PT) and prostate cancer metastases (MD) by RT-PCR. $H_2O$ included as negative control. (**A**) Fragments from the 3′ end of the No55 open reading frame were amplified by RT-PCR, blotted and hybridized to a $^{32}$P-labelled fragment from No55 cDNA (top panel). β-actin expression visualized on an ethidium bromide stained agarose gel (lower panel). (**B**) Fragments covering the No55 open reading frame were amplified by RT-PCR, blotted and hybridized to a $^{32}$P-labelled fragment from No55 cDNA (top panel). S9 expression visualized on an ethidium bromide stained agarose gel (lower panel)

(TAT C**A**G GGG) and DU145 (TAT C**G**G GGG) were found, indicative of an allelic polymorphism this position. In patient MD1 all four clones were identical to the T24 sequence (TAT C**A**G GGG). Other deviations from the published No55 ORF were not found, neither in testis nor in tumour material.

### Expression pattern of No55 in normal tissues and cell lines

Northern analysis of No55 in normal human tissues is depicted in Figure 1. A 2.6 kb transcript was detected with the strongest signals in ovaries, pancreas and prostate and weaker signals in testis, heart, kidney colon, spleen and placenta. Quantified expression levels were similar in ovaries and pancreas, 2-fold higher for ovaries relative to prostate and 8-fold higher in ovaries relative to placenta. No expression above background was recorded for the other human tissues tested. Expression of No55 was also found in three additional prostate cancer cell lines (PC-3, PC-3m and LNCaP) (Figure 2).

### Expression of No55 in biopsies from normal prostate tissue and prostate cancer

Due to the low amount of tissue available, expression of No55 in biopsies from normal prostate tissue, primary prostate tumours and metastatic lesions was analysed by semi-quantitative RT-PCR (Figure 3A). Expression was found in five out five biopsies from normal prostate tissue and six out of six primary tumour biopsies at varying levels. Two primary tumours expressed No55 at levels 3–4 times higher than the average level for normal prostate tissue. Expression in the four remaining samples was similar to normal tissue using a 3-fold increase relative to the normal prostate tissue samples as a cut-off value. For two metastatic lesions (from the seroreactive patient MD1 and the seronegative patient MD25), No55 expression was found to be 4- and 5-fold higher, respectively, compared to normal prostate tissue.

In a second set of experiments the complete No55 ORF was amplified, and the expression level compared in matched biopsies from normal prostate tissue and primary tumours from individual patients and metastatic tissue from other patients (Figure 3B). For the three paired samples, no significant increase in expression was detected in primary tumours compared to normal tissue. Again, expression in the two metastatic lesions (from patients MD1 and MD25) was found to be increased 5- and 6-fold, respectively, compared to the average of the three normal prostate tissue samples.

### Screening for anti-No55 antibodies in sera from patients with prostate cancer and healthy controls

A plaque assay was used to test for IgG antibodies against No55 protein in sera of patients with prostate cancer and healthy male volunteers. Preabsorbed sera were assayed in a dilution of 1:100. Among the 45 patients with local disease, six had detectable anti-No55 antibodies, as had one out of two patients with metastatic disease. Altogether, a positive result was obtained for seven out of 47 patients (15%). None of the 20 healthy male controls had detectable anti-No55 antibodies.

© 2000 Cancer Research Campaign

### Chromosomal localization of the gene encoding No55

Southern analysis of DNA from peripheral blood leukocytes and cell lines DU145, Hela and Reh indicated that the No55 gene is a single copy gene and gave no evidence for amplification of the gene in cell lines with a high No55 expression (data not shown). Radiation hybrid mapping with an STS derived from the No55 cDNA sequence placed the No55 gene on the long arm of chromosome 17 in close proximity (distance 7cR, LOD-Score 10.47) to the STS SHGC-30259. This latter STS located in the interval between D17S800 and D17S930 (62.9–64.3 cM) at 2171 cR10 000 (F) on the GeneMap99 RH map is specific for the human JUP (Junction plakoglobin) gene. The JUP gene has been cytogenetically assigned to the chromosomal region 17q21 (http://www.ncbi.nlm.nih.gov/cgi-bin/UniGene). In line with this location, PAC 228H19 specific for the No55 STS was mapped to 17q21 by FISH and subsequent R-banding. Further support for the location of No55 on chromosome 17 comes from sequence comparisons using the BLASTN algorithm, that revealed the human chromosome 17 specific BAC-clone RP11-156E6 (AC012192) to contain the No55 gene.

### DISCUSSION

In the present study sera from prostate cancer patients were used to identify tumour-associated antigens expressed in a prostate cancer cell line. The choice of cell line DU145 and sera was based on the following rationale. DU145 is derived from a prostate cancer metastasis, and thus potentially expresses a spectrum of antigens representative of advanced disease. Sera used for screening included patients with limited disease, to isolate antigens with relevance to early stages of the disease. Thereby, it was expected to increase the yield of antigens that are present in early stages of the disease and that are retained throughout tumour progression.

The yield of immunoreactive proteins in the present study appears to be lower than reported in studies of SEREX in other malignancies. Using cDNA libraries from autologous tumour, testis tissue or cell lines, a larger number of different immunogenic proteins are found per patient serum. To our knowledge, the four different genes isolated in this study did not correspond to any of the genes isolated by other investigators, and did not match any sequences released in the publicly available part of the SEREX database (*http://www-ludwig.unil.ch/SEREX*). These differences may result from the experimental design, i.e. the use of allogeneic sera from early-stage patients and a cell line cDNA library. This could indicate that the number of immunogenic proteins retained in the tumour throughout progression may be low. There may also be differences in the immunobiology of prostate cancer and other types of cancer.

Of the four genes identified by immunoscreening, the gene encoding the nucleolar protein No55 was further analysed. No55 was initially described as a 55 kD nucleolar protein in human cell lines recognized by IgG antibodies in the serum of a female patient with interstitial cystitis (IC) (Ochs et al, 1996). The cDNA encoding the No55 protein was cloned by immunoscreening of an expression library from the bladder carcinoma cell line T24 and contained an ORF corresponding to a predicted molecular mass of 50 kD (Ochs et al, 1996). The primary structure of the No55 protein reveals 87% sequence identity and 92% sequence similarity to SC65, a protein isolated from the synaptonemal complexes (SC) of meiotic chromosomes of rat testicles (Chen et al, 1992).

No55 is expressed in several normal human tissues. The highest mRNA expression levels were detected in ovaries, pancreas and prostate and lower levels in other tissues. Several tissues tested did not reveal detectable No55 transcripts by Northern blot analysis. This expression pattern in human tissues is markedly different from the expression pattern of the homologous protein SC65 in rats. Rat SC65 mRNA was detected at the highest levels in testis, brain and heart, and a lower level in liver (Chen et al, 1992). However, it cannot be excluded that more human tissues express No55 mRNA at low levels not detectable by Northern blot analysis.

No55 expression could be demonstrated in all analysed prostate cancer cell lines and tumour samples from prostate cancer, including primary tumours and metastatic tissue. No55 expression thus does not appear to be lost during malignant transformation of prostate epithelial cells and subsequent tumour growth. Based on the standardization by mRNAs for β-actin or ribosomal S9 protein used for the semi-quantitative approach, there was clear over-expression of No55 in metastatic lesions. Although a subset of primary tumour biopsies contained No55 transcripts at levels considerably higher than the average of normal prostate tissue samples, no clear difference in expression level was found for three sets of matched biopsies from primary tumours and normal prostate tissue from the same individuals. A firm conclusion on the expression level of No55 in primary tumours is hampered for several reasons. First, the standardization of RT-PCR experiments using housekeeping genes such as β-actin or S9 is not reliable in all cases, as variations in expression levels of these genes do occur (Spanakis and Brouty-Boye, 1994). Secondly, the relative amounts of stroma cells may vary between biopsy specimens. In the present study, morphological analysis of all samples used for RNA extraction showed differences in the amount of non-epithelial cells, comparing both different samples and different areas of each individual sample. The availability of a specific antibody for use in immunohistochemistry will potentially allow a better assessment of the relative expression levels of No55 in metastatic lesions, primary tumours and normal prostate tissue.

The function of No55 in tumour cells is unknown. However, No55 displays similarities to SCP-1, a member of the SEREX-defined cancer/testis family of tumour antigens (Tureci et al, 1998). The SCP-1 protein is localized in the SC of meiotic chromosomes in human testicles and in the nucleus of malignant cells. It has been proposed that ectopic expression of SCP-1 may interfere with chromosome segregation during mitosis (Tureci et al, 1998). Similarly, SC65, the rat homologue of No55, has been shown to be localized in the SC and No55 is expressed in ovaries and testicles, both organs involved in gametogenesis. In analogy, one may speculate that No55 could contribute to mitotic instability. No55 expression may also be related to the proliferative activity of somatic cells. Other nucleolar antigens, such as p120 and p145, have been found to be associated with proliferating cells of normal tissues and to be expressed in a variety of malignant tumours (Freeman et al, 1989). Both proteins have been shown to be of relevance to prostate cancer and p120 appears to be an additional prognostic marker for carcinoma of the prostate (Qiao et al, 1994; Kallakury et al, 1999). Further work is required in order to establish the function of No55 in cancer cells in general and carcinoma of the prostate in particular.

There is considerable overlap in the repertoire of autoantibodies found in patients with autoimmune disease and cancer patients

© 2000 Cancer Research Campaign

(Gure et al, 1998). Interestingly, No55 was originally cloned using serum from a patient with IC, a condition where autoimmune destruction of the bladder wall is believed to be play a role in the pathogenesis (Theoharides et al, 1998). However, little is known about the incidence of anti-No55 antibodies in patients with IC or autoimmune disease in general. Autoantibodies with a nucleolar staining were found in 7% of patients with IC, but the specificities of the individual patients' antibodies have not been reported (Ochs et al, 1994). To the best of our knowledge, anti-No55 antibodies have not been described in patients with other autoimmune disease or in normal controls. In contrast, autoantibodies of the IgG subclass against No55 were found in 15% of prostate cancer patients in the present study and none of the seven patients with anti-No55 antibodies identified had a history of IC or other autoimmune disease. However, the finding of anti-No55 antibodies in patients with IC raises concern as to potential use of No55 as a target antigen in immunotherapy of prostate cancer.

No direct evidence presented in this paper indicates that No55 is mutated in prostate cancer. The only sequence differences identified in the ORF probably result from an allelic polymorphism. However, the No55 gene is located at position 17q21, a locus reported to be partially deleted in 50% of prostate cancers (Gao et al, 1995). A tumour suppressor gene (TSG) with relevance to the development of prostate cancer has been suspected in this region and BRCA-1 is a candidate TSG at this locus (Murakami et al, 1995; Fan et al, 1998; Uchida et al, 1999). Whether deletions in region 17q21 in prostate cancers affect noncoding regions of the No55 gene, i.e. regulatory regions of gene expression, remains to be elucidated.

Three genes isolated in the present study, TDP-43, Drebrin and $PP4_R$, have not yet been analysed in detail although the corresponding proteins prove to be immunogenic in individual prostate cancer patients. Drebrin is differentially expressed during neuronal development and, through binding to actin filaments, the protein is involved in neuronal morphogenesis (Shirao, 1995). The cloning of Drebrin by SEREX may relate to the neuroendocrine differentiation reported for DU145 and the fact that approximately 50% of prostate cancer patients show neuroendocrine elements in their tumours (Cussenot et al, 1998; Hoosein, 1998). $PP4_R$ has recently been cloned but its function in human tissues is not clear (Kloeker and Wadzinski, 1999). $PP4_R$ is broadly expressed in human tissues including the prostate gland and the $PP4_R$ primary sequence contains 13 non-identical repeats of approximately 40 amino acids in length. Repetitive elements in the primary structure have been found as a predominant feature in other tumour antigens recognized by SEREX (Gure et al, 1998).

In conclusion, among four immunogenic proteins identified by serological analysis, No55 displays a restricted expression profile in normal and malignant tissues, is expressed in early and late stages of prostate cancer and is immunogenic in a subset of patients. These data, together with the chromosomal localization of the No55 gene, warrant further investigation of the potential role of No55 in the biology and tumour immunology of prostate cancer.

### ACKNOWLEDGEMENTS

This work was supported by the Norwegian Cancer Society.

### REFERENCES

Chen Q, Pearlman RE and Moens PB (1992) Isolation and characterization of a cDNA encoding a synaptonemal complex protein. *Biochem Cell Biol* **70**: 1030–1038

Chen YT, Scanlan MJ, Sahin U, Tureci O, Gure AO, Tsang S, Williamson B, Stockert E, Pfreundschuh M and Old LJ (1997) A testicular antigen aberrantly expressed in human cancers detected by autologous antibody screening. *Proc Natl Acad Sci USA* **94**: 1914–1918

Chen YT, Gure AO, Tsang S, Stockert E, Jager E, Knuth A and Old LJ (1998) Identification of multiple cancer/testis antigens by allogeneic antibody screening of a melanoma cell line library. *Proc Natl Acad Sci USA* **95**: 6919–6923

Chomczynski P and Sacchi N (1987) Single-step method of RNA isolation by acid guanidinium thiocyanate-phenol-chloroform extraction. *Anal Biochem* **162**: 156–159

Cussenot O, Villette JM, Cochand-Priollet B and Berthon P (1998) Evaluation and clinical value of neuroendocrine differentiation in human prostatic tumours. *Prostate Suppl* **8**: 43–51

Fan S, Wang JA, Yuan RQ, Ma YX, Meng Q, Erdos MR, Brody LC, Goldberg ID and Rosen EM (1998) BRCA1 as a potential human prostate tumour suppressor: modulation of proliferation, damage responses and expression of cell regulatory proteins. *Oncogene* **16**: 3069–3082

Freeman JW, Hazlewood JE, Bondada V, Cibull ML, Fonagy A, Ochs R, Busch RK and Busch H (1989) Proliferation-related nucleolar antigens P145 and P120 associated with separate nucleolar elements and differences in tissue distribution. *Cancer Commun* **1**: 367–372

Gao X, Zacharek A, Grignon DJ, Sakr W, Powell IJ, Porter AT and Honn KV (1995) Localization of potential tumour suppressor loci to a <2 Mb region on chromosome 17q in human prostate cancer. *Oncogene* **11**: 1241–1247

Gure AO, Altorki NK, Stockert E, Scanlan MJ, Old LJ and Chen YT (1998) Human lung cancer antigens recognized by autologous antibodies: definition of a novel cDNA derived from the tumour suppressor gene locus on chromosome 3p21.3. *Cancer Res* **58**: 1034–1041

Harder J, Siebert R, Zhang Y, Matthiesen P, Christophers E, Schlegelberger B and Schroder JM (1997) Mapping of the gene encoding human beta-defensin-2 (DEFB2) to chromosome region 8p22–p23.1. *Genomics* **46**: 472–475

Hoosein NM (1998) Neuroendocrine and immune mediators in prostate cancer progression. *Front. Biosci* **3:D1274-9**: D1274–D1279

Itoh M, Watanabe M, Yamada Y, Furukawa K, Taniguchi M, Hata T, Schmitt M, Ikeda H, Yamaguchi M, Ohno T, Nakashima K and Shiku H (1999) HUBI is an autoantigen frequently eliciting humoral immune response in patients with adult T cell leukemia. *Int J Oncol* **14**: 703–708

Jager E, Chen YT, Drijfhout JW, Karbach J, Ringhoffer M, Jager D, Arand M, Wada H, Noguchi Y, Stockert E, Old LJ and Knuth A (1998) Simultaneous humoral and cellular immune response against cancer-testis antigen NY-ESO-1: definition of human histocompatibility leukocyte antigen (HLA)-A2-binding peptide epitopes. *J Exp Med* **187**: 265–270

Kallakury BV, Sheehan CE, Rhee SJ, Fisher HA, Kaufman RPJ, Rifkin MD and Ross J S (1999) The prognostic significance of proliferation-associated nucleolar protein p120 expression in prostate adenocarcinoma: a comparison with cyclins A and B1, Ki-67, proliferating cell nuclear antigen, and p34cdc2. *Cancer* **85**: 1569–1576

Kawakami Y and Rosenberg SA (1997) Human tumour antigens recognized by T-cells. *Immunol Res* **16**: 313–339

Kloeker S and Wadzinski BE (1999) Purification and identification of a novel subunit of protein serine/threonine phosphatase 4. *J Biol Chem* **274**: 5339–5347

Murakami YS, Brothman AR, Leach RJ and White RL (1995) Suppression of malignant phenotype in a human prostate cancer cell line by fragments of normal chromosomal region 17q. *Cancer Res* **55**: 3389–3394

Ochs RL, Stein TWJ, Chan EK, Ruutu M and Tan EM (1996) cDNA cloning and characterization of a novel nucleolar protein. *Mol Biol Cell* **7**: 1015–1024

Ochs RL, Stein TWJ, Peebles CL, Gittes RF and Tan EM (1994) Autoantibodies in interstitial cystitis. *J Urol*. **151**: 587–592

Old LJ and Chen YT (1998) New paths in human cancer serology. *J Exp Med* **187**: 1163–1167

Ou SH, Wu F, Harrich D, Garcia-Martinez LF and Gaynor RB (1995) Cloning and characterization of a novel cellular protein, TDP-43, that binds to human immunodeficiency virus type 1 TAR DNA sequence motifs. *J Virol* **69**: 3584–3596

Qiao L, Pizzolo JG and Melamed MR (1994) Effects of suramin on expression of proliferation associated nuclear antigens in DU-145 prostate carcinoma cells. *Biochem Biophys Res Commun* **201**: 581–588

Rosok O, Pedeutour F, Ree AH and Aasheim HC (1999) Identification and characterization of TESK2, a novel member of the LIMK/TESK family of protein kinases, predominantly expressed in testis. *Genomics* **61**: 44–54

Sahin U, Tureci O, Schmitt H, Cochlovius B, Johannes T, Schmits R, Stenner F, Luo G, Schobert I and Pfreundschuh M (1995) Human neoplasms elicit multiple specific immune responses in the autologous host. *Proc Natl Acad Sci USA* **92**: 11810–11813

 © 2000 Cancer Research Campaign

Scanlan MJ, Chen YT, Williamson B, Gure AO, Stockert E, Gordan JD, Tureci O, Sahin U, Pfreundschuh M and Old LJ (1998) Characterization of human colon cancer antigens recognized by autologous antibodies. *Int J Cancer* **76**: 652–658

Scanlan MJ, Gordan JD, Williamson B, Stockert E, Bander NH, Jongeneel V, Gure A O, Jager D, Jager E, Knuth A, Chen YT and Old LJ (1999) Antigens recognized by autologous antibody in patients with renal-cell carcinoma. *Int J Cancer* **83**: 456–464

Shirao T (1995) The roles of microfilament-associated proteins, drebrins, in brain morphogenesis: a review. *J Biochem (Tokyo)* **117**: 231–236

Spanakis E and Brouty-Boye D (1994) Evaluation of quantitative variation in gene expression. *Nucleic Acids Res* **22**: 799–806

Theoharides TC, Pang X, Letourneau R and Sant GR (1998) Interstitial cystitis: a neuroimmunoendocrine disorder. *Ann N Y Acad Sci* **840**: 619–634

Toda M, Shirao T, Minoshima S, Shimizu N, Toya S and Uyemura K (1993) Molecular cloning of cDNA encoding human drebrin E and chromosomal mapping of its gene. *Biochem Biophys Res Commun* **196**: 468–472

Tureci O, Sahin U, Zwick C, Koslowski M, Seitz G and Pfreundschuh M (1998) Identification of a meiosis-specific protein as a member of the class of cancer/testis antigens. *Proc Natl Acad Sci USA* **95**: 5211–5216

Uchida T, Wang C, Sato T, Gao J, Takashima R, Irie A, Ohori M and Koshiba K (1999) BRCA1 gene mutation and loss of heterozygosity on chromosome 17q21 in primary prostate cancer. *Int J Cancer* **84**: 19–23

*© 2000 Cancer Research Campaign*
